# Orthoptic Treatment After Strabismus Surgery in Child Intermittent Divergent Strabismus

**DOI:** 10.3390/children13010070

**Published:** 2026-01-01

**Authors:** Pedro Lino, Pedro Vargues de Aguiar, João Paulo Cunha

**Affiliations:** 1 NOVA National School of Public Health, Public Health Research Centre, Comprehensive Health Research Center, CHRC, LA-REAL, CCAL, NOVA University Lisbon, 1649-004 Lisbon, Portugal; 2 CUF Cascais Hospital, 2750-663 Cascais, Portugal; 3 CUF Tejo Hospital, 1350-352 Lisbon, Portugal; 4 Scientific Area of Orthoptics and Vision Sciences, Higher School of Technology and Health of Lisbon, Polytechnic Institute of Lisbon, 1990-096 Lisbon, Portugal; 5 Ophthalmology Unit, CUF Cascais Hospital, 2750-663 Cascais, Portugal; 6 Department of Morpho-Functional Sciences, Higher School of Technology and Health of Lisbon, Polytechnic Institute of Lisbon, 1990-096 Lisbon, Portugal

**Keywords:** convergence insufficiency, intermittent exotropia, orthoptic therapy

## Abstract

**Highlights:**

**What are the main findings?**

- Postoperative orthoptic therapy producedlarge improvements in sensory–motor function, including fusional amplitudes and convergence measures (NPC and convergence amplitudes).
- Static ocular alignment changed only minimally, showing a small exo-drift consistent with the normal postoperative course rather than a therapeutic effect.

**What is the implication of the main finding?**

- Orthoptic therapyenhances binocular stability and functional controlafter strabismus surgery, even when ocular alignment itself remains largely unchanged.
- These sensory–motor gains maysupport long-term binocular function and reduce the risk of postoperative decompensation or recurrencein children with intermittent exotropia.

**Abstract:**

**Purpose:** To evaluate short-term motor and sensory–motor outcomes following postoperative OT in children with IXT after strabismus surgery. **Methods:** This prospective before–after observational study included children with IXT who underwent bilateral lateral rectus recession and were referred for postoperative OT based on predefined clinical criteria. A structured 12-week OTplan was initiated approximately six months after surgery. Outcome measures included angle of deviation (prism diopters, PD), near point of convergence (cm), positive fusional vergence amplitudes (PD), and convergence amplitudes at distance and near (PD). Pre- and post-therapy changes were analysed using paired-samples *t*-tests with effect sizes calculated using Cohen’s d. Final postoperative alignment was additionally compared cross-sectionally between children who underwent OT and those managed without OT. **Results:** Eighty-eight children had complete paired motor and sensory–motor data and were included in the analyses. Changes in static ocular alignment were small, with mean residual deviation improving from −7.02 ± 6.91 PD to −5.22 ± 6.60 PD after OT (mean change +1.80 PD; *p* < 0.01; d ≈ 0.30). No significant difference in final postoperative alignment was observed between the OT and non-OT groups (*p* = 0.827). In contrast, marked improvements were observed in sensory–motor outcomes. Positive fusional vergence amplitude increased from 7.30 ± 8.33 PD to 22.19 ± 9.26 PD (*p* < 0.001; d ≈ 1.5). Distance convergence amplitude improved from 7.30 ± 8.33 PD to 22.19 ± 9.26 PD, and near convergence amplitude from 10.95 ± 12.50 PD to 33.29 ± 13.89 PD (both *p* < 0.001; d ≈ 1.5). Near point of convergence showed a modest but significant improvement. **Conclusions:** Postoperative OT was associated with substantial short-term improvements in sensory–motor function, particularly fusional and convergence capacities, while changes in static ocular alignment were small and of limited clinical relevance. These findings support the role of OT as a functional adjunct to surgery, aimed at enhancing binocular control and postoperative sensory–motor stability in children with IXT.

## 1. Introduction

Intermittent exotropia (IXT) is one of the most common forms of childhood strabismus, accounting for up to 25% of pediatric strabismus cases and affecting approximately 1% of the general population, although prevalence varies across geographic regions and ethnic groups [1,2]. It is characterized by alternating periods of well-controlled exophoria and manifest exotropia, reflecting an unstable balance between motor alignment and binocular sensory control. Due to its intermittent nature, the clinical course of IXT is heterogeneous and unpredictable, and management strategies range from observation to surgical and non-surgical interventions.

The etiology of IXT remains incompletely understood. Unlike restrictive or paralytic strabismus, IXT is a comitant deviation with preserved extraocular muscle structure and function. Increasing evidence supports a predominantly neurophysiological basis, involving altered cortical and subcortical mechanisms of binocular integration and vergence control [3,4]. Functional imaging and experimental studies have implicated dysfunction within cortical areas responsible for binocular fusion, as well as cerebellar and brainstem pathways involved in vergence adaptation [5]. These findings suggest that impaired sensory–motor integration, rather than a primary mechanical abnormality, plays a central role in the development and persistence of IXT.

Surgical treatment aims to restore ocular alignment by modifying extraocular muscle tension and remains the standard approach for patients with poorly controlled or progressive exotropia. However, postoperative motor alignment alone does not guarantee stable binocular function. Several longitudinal studies have demonstrated that a proportion of children experience postoperative exo-drift over time, even after initially successful surgery [6,7,8]. This phenomenon highlights the importance of postoperative sensory–motor adaptation and the potential need for adjunctive therapies to reinforce binocular stability.

Orthoptic therapy (OT) is designed to target neurosensory mechanisms underlying binocular vision, including convergence ability, fusional vergence reserves, suppression control, and binocular cooperation. Preoperative orthoptic interventions have been shown to improve symptoms and fusional capacity in selected cases, particularly in near-dominant IXT associated with convergence insufficiency [9]. Postoperatively, OThas been proposed to enhance vergence control and stabilize binocular function after surgical realignment.

Several studies have suggested that combining surgery with OT may improve functional outcomes compared with surgery alone [10,11,12]. Randomized and non-randomized studies have reported improvements in stereoacuity, fusional reserves and control scores in patients receiving postoperative binocular training [11,12]. Nevertheless, the available evidence remains heterogeneous, with substantial variability in study design, timing of intervention, therapeutic protocols, and outcome measures. Short-term postoperative functional data obtained using structured and standardized orthoptic protocols remain limited.

Moreover, many previous studies have focused primarily on motor outcomes or long-term recurrence rates, with less emphasis on early postoperative sensory–motor changes that may be clinically relevant for visual comfort and functional binocular control. Differentiating between motor alignment, sensory outcomes and fusional control is essential to clarify the specific contribution of postoperative OT.

In this context, the present study aimed to evaluate the short-term impact of a structured postoperative OTplan on sensory–motor outcomes in children who had undergone surgery for IXT. Specifically, we assessed changes in near point of convergence (NPC), positive fusional amplitudes and angle of deviation, and compared these outcomes with those observed in a matched non-treated postoperative cohort, to better characterize the functional role of orthoptic rehabilitation in the early postoperative period.

## 2. Methodology

Study Design and Participants: This prospective before–after observational study included pediatric patients diagnosed with IXT who underwent surgical correction followed by postoperative orthoptic assessment at a single tertiary referral centre between January 2015 and December 2025. A total of 256 children who had undergone strabismus surgery for IXT were initially identified from the institutional database.

Postoperative OT was offered to children presenting residual deviation or exophoria, reduced fusional control, or impaired stereopsis, provided they were cooperative and able to perform the prescribed exercises. Eligibility for orthoptic intervention required residual deviations within surgically acceptable limits (≤15 DP). The primary aims of postoperative OTwere to enhance motor control and reinforce binocular fusion. Therapeutic exercises were individualized, with a primary focus on fusion and convergence training to optimize postoperative binocular function.

Of the total cohort, 97 children were referred for postoperative OT based on these predefined clinical criteria. Eighty-eight of these patients had complete paired measurements of ocular alignment and sensory–motor outcomes and were included in the paired analyses. The remaining patients did not undergo postoperative OTand were followed according to routine clinical practice.

Eligible participants had undergone bilateral lateral rectus recession for IXT and had available postoperative clinical records. Exclusion criteria included paralytic or restrictive strabismus, previous strabismus surgery, significant ocular pathology other than refractive error, neurological disorders affecting binocular vision, and incomplete clinical data.

The study was conducted in accordance with the tenets of the Declaration of Helsinki. Written informed consent was obtained from the parents or legal guardians of all participants.

For patients who did not undergo postoperative OT, the angle of deviation at final postoperative follow-up was extracted from clinical records and used for cross-sectional comparison with the OT group. No longitudinal paired sensory–motor data were available for this cohort.

Surgical Procedure:All patients underwent bilateral lateral rectus recession performed by the same surgical team using a standardized surgical technique. The amount of recession was determined according to the preoperative angle of deviation and ranged from a total recession of 10 mm (5 mm per eye) to 18 mm (9 mm per eye). All procedures were performed under general anesthesia following institutional protocols. No patient required reoperation during the study period.

Orthoptic Therapy Protocol: Postoperative OTwas initiated approximately six months after surgery, once ocular alignment had stabilized, and no residual postoperative inflammation was present. Referral for OTwas not randomized and was based on predefined clinical criteria, as described above.

The OTplan had a fixed duration of 12 weeks for all patients. Therapy consisted of one supervised in-clinic session per week, lasting approximately 30–40 min, conducted by a certified orthoptist experienced in pediatric binocular vision rehabilitation. In addition, patients were prescribed a complementary home-based exercise plan to be performed daily for approximately 10–15 min under parental supervision.

Therapeutic interventions were designed to enhance convergence ability, improve positive fusional vergence amplitudes, and promote stable binocular sensory integration. Convergence training included accommodative and non-accommodative exercises such as pencil push-ups, near–far fixation shifts, and step convergence tasks. Fusional vergence training was performed using base-out prism activities with progressive increases in demand according to patient tolerance and clinical response.

In-clinic sensory fusion and anti-suppression training were systematically performed using a synoptophore. These exercises addressed simultaneous perception, fusion, and stereopsis, with gradual adjustments in angular separation to stimulate fusional reserves and reduce suppression when present.

Optical Correction and Cycloplegic Refraction:All assessments and therapy sessions were performed with full optical correction. Cycloplegic refraction was conducted using tropicamide 1% (10 mg/mL), instilled twice at a 5min interval. Static retinoscopy was performed 20 min after the final instillation, and the prescribed optical correction corresponded to the measured refractive error without over- or undercorrection.

Outcome Measures:Ocular alignment was assessed in prismbars (PD) using standard clinical prism cover testing. Negative values denote exodeviation.

The near point of convergence was measured using a standard RAF rule under controlled clinical conditions.

Fusional vergence amplitudes in free space were assessed using a Berens prism bar and were recorded directly in PD. Sensory fusion and vergence training performed on the synoptophore yielded measurements in degrees; when reported, these values were converted to PDusing a standard conversion factor (1° = 1.75 PD) to ensure consistency across outcome measures.

Exo-drift was defined as a postoperative shift toward increasing exodeviation, corresponding to more negative PD values over time.

Statistical Analysis: Statistical analyses were performed using SPSS software (V29), IBM Corp., Armonk, NY, USA). Continuous variables were expressed as mean ± standard deviation, and categorical variables as absolute frequencies and percentages.

Within-group pre- and post-therapy comparisons in the OT group were analyzed using paired-samples *t*-tests. Effect sizes were calculated using Cohen’s d to quantify the magnitude of observed changes. A two-tailed *p*-value of less than 0.05 was considered statistically significant.

A cross-sectional comparison of final postoperative alignment between the OT and non-OT groups was performed using an independent-samples *t*-test with Welch correction when variance homogeneity was not assumed.

## 3. Results

### 3.1. Study Sample

From the initial cohort of 256 children who underwent surgery for IXT, 97 were referred for postoperative OT based on predefined clinical criteria. Of these, 88 children had complete paired pre- and post-therapy measurements and were included in the paired analyses of motor and sensory–motor outcomes.

The remaining patients did not undergo postoperative OT and were followed according to routine clinical practice. Data from this group were used descriptively to contextualize postoperative residual deviation but were not included in longitudinal paired analyses.

### 3.2. Sample

Out of the total dataset (n = 256), 90 patients received OT and 160 served as potential controls. See Table 1.

### 3.3. Angle of Deviation

In the OT group, a small but statistically significant change in ocular alignment was observed over the treatment period. Mean residual deviation changed from −7.02 ± 6.91 PD at baseline to −5.22 ± 6.60 PD after completion of orthoptic therapy, corresponding to a mean reduction in exodeviation of approximately 1.8 PD (paired analysis, *p* < 0.01; Cohen’s d ≈ 0.30).

According to the predefined sign convention, this change represents a slight reduction in exodeviation rather than postoperative exo-drift. The magnitude of this change was small, indicating that postoperative OTdid not substantially modify static ocular alignment beyond expected postoperative variability.

### 3.4. Near Point of Convergence

Near point of convergence improved following postoperative orthoptic therapy. Mean NPC decreased from 9.45 ± 4.13 cm at baseline to 8.15 ± 1.84 cm after therapy, representing a mean improvement of 1.30 cm (*p* < 0.01; Cohen’s d ≈ −0.36). This finding indicates enhanced convergence efficiency afternear completion of the therapy plan.

### 3.5. Positive Fusional Amplitude

OT produced a robust and clinically meaningful improvement in fusional reserves. Positive fusional amplitude increased from 4.17 ± 4.76° at baseline to 12.68 ± 5.29° after therapy (mean change +8.51 ± 5.64°; *p* < 0.001; Cohen’s d = 1.51), representing a large effect size. This finding demonstrates substantial enhancement in the ability to maintain single binocular vision under increased fusional demand.

### 3.6. Convergence Amplitudes

Postoperative OT was associated with a marked improvement in positive fusional vergence amplitudes. Mean positive fusional amplitude increased from 7.30 ± 8.33 PD at baseline to 22.19 ± 9.26 PD after therapy, corresponding to a mean increase of 14.89 ± 9.87 PD (*p* < 0.001; Cohen’s d ≈ 1.5), representing a large effect size.

Significant improvements were also observed in convergence amplitudes measured in free space.

Distance convergence amplitude increased from 7.30 ± 8.33 PD at baseline to 22.19 ± 9.26 PD after therapy (*p* < 0.001; Cohen’s d≈ 1.5).

Near convergence amplitude increased from 10.95 ± 12.50 PD to 33.29 ± 13.89 PD following therapy (*p* < 0.001; Cohen’s d ≈ 1.5).

These findings indicate a substantial strengthening of convergence control mechanisms following the structured postoperative OT plan.

Table 2 summarizes the pre- and post-therapy changes in ocular alignment, convergence function, and fusional vergence observed in children undergoing postoperative orthoptic therapy. While changes in static ocular alignment were modest, large and clinically meaningful improvements were observed in sensory–motor parameters, particularly fusional reserves and convergence amplitudes.

### 3.7. Final Postoperative Alignment: Orthoptic Therapy vs. No Therapy

At final postoperative follow-up, the angle of deviation was compared cross-sectionally between children who underwent postoperative orthoptic therapy and those who did not. An independent-samples *t*-test with Welch correction was used due to unequal variances between groups.

No statistically significant difference in final ocular alignment was observed between the orthoptic therapy group and the non-therapy group (mean difference −2.77 DP, *p* = 0.827). The wide confidence interval reflected the small sample size and variability within the non-therapy group.

These findings indicate that final postoperative alignment was comparable between children who received postoperative orthoptic therapy and those managed with routine follow-up alone.

Although a greater mean exodeviation at final follow-up was observed in the non-OT group, a formal statistical assessment of postoperative exo-shift within this group could not be performed due to the absence of longitudinal paired alignment data.

## 4. Discussion of Results

The present study investigated the short-term effects of a structured postoperative OT plan on motor and sensory–motor outcomes in children surgically treated for intermittent exotropia. The findings demonstrate that postoperative OT was associated with substantial improvements in sensory–motor function—particularly positive fusional vergence and convergence amplitudes—while changes in static ocular alignment were small and of limited clinical relevance.

Regarding motor alignment, only a modest reduction in exodeviation was observed within the OTgroup, amounting to approximately 1.8 DP. Although statistically significant, this change is unlikely to represent a clinically meaningful modification of the mechanical alignment achieved by surgery. According to the predefined sign convention, this change reflected a slight reduction in exodeviation rather than postoperative exo-drift. These results are consistent with previous studies indicating that postoperative ocular alignment in intermittent exotropia is primarily determined by surgical factors and postoperative motor adaptation, with limited influence from vergence-based training alone [6,7].

To address concerns regarding the absence of a control group, a cross-sectional comparison of final postoperative alignment was performed between children who underwent orthoptic therapy and those who did not. No statistically significant difference in residual deviation was observed between the two groups at final follow-up. This finding must be interpreted cautiously due to the small sample size and heterogeneity of the non-therapy group, as reflected by the wide confidence intervals and limited statistical power. Nevertheless, it is noteworthy that children referred for orthoptic therapy—who typically presented with poorer postoperative sensory–motor profiles—achieved final ocular alignment comparable to those managed with routine follow-up alone. This observation does not support a causal effect of orthoptic therapy on motor alignment but suggests that postoperative orthoptic intervention does not compromise surgical outcomes and may contribute to functional stabilization in clinically more vulnerable patients.

In contrast to the limited changes observed in static alignment, sensory–motor outcomes showed marked and clinically meaningful improvements following orthoptic therapy. Positive fusional vergence amplitudes increased substantially, with large effect sizes, indicating a significant enhancement in the ability to maintain binocular fusion under increasing vergence demand. Reduced fusional reserves are a well-recognized feature of intermittent exotropia and have been associated with poor control, visual symptoms, and postoperative instability [5,8]. Strengthening fusional capacity may therefore play a key role in improving functional binocular stability, even when static ocular alignment remains largely unchanged.

Similarly, convergence amplitudes at both distancesand near improved significantly after therapy, again with large effect sizes. These findings align with previous reports demonstrating that structured vergence and binocular training can induce meaningful improvements in convergence performance and binocular coordination [9,11]. Such improvements are thought to reflect adaptive plasticity within cortical and subcortical pathways involved in vergence control and binocular integration, rather than changes at the level of the extraocular muscles [3,4,10].

The improvement observed inthenear point of convergence, although more modest in magnitude, was statistically significant and clinically relevant. NPC is a sensitive indicator of convergence efficiency and is closely related to visual comfort and near-task performance in children [9]. Even small improvements in NPC may reduce asthenopia symptoms and enhance functional performance during sustained near activities, particularly in the postoperative period when binocular control mechanisms are still adapting.

Taken together, these findings support the concept that postoperative orthoptic therapy primarily enhances functional binocular control rather than substantially altering the mechanical alignment achieved surgically. This distinction is clinically important and underscores the complementary roles of surgery and orthoptic rehabilitation in the management of intermittent exotropia: surgery restores ocular alignment, while orthoptic therapy appears to consolidate sensory–motor integration and strengthen the patient’s ability to maintain binocular single vision under physiological visual demands.

Several limitations of this study should be acknowledged. The observational before–after design and the non-randomized referral to postoperative OT introduce the potential for selection bias. In addition, longitudinal paired sensory–motor and alignment data were not available for the non-OT group, limiting formal statistical assessment of postoperative exo-drift and causal inference regarding the preventive effect of OT. A further limitation is the absence of objective quantitative monitoring of adherence to the home-based component of the OT plan. Although all patients and caregivers received standardized written and verbal instructions, and compliance was routinely reinforced during supervised in-clinic sessions, adherence to daily home exercises was not formally recorded. Consequently, inter-individual variability in compliance cannot be excluded and may have influenced the magnitude of the observed sensory–motor improvements. Finally, the relatively short follow-up period precludes conclusions regarding the long-term durability of these functional gains and their impact on recurrence rates.

Future prospective randomized controlled studies with longer follow-up periods are warranted to determine whether the short-term sensory–motor gains associated with postoperative orthoptic therapy translate into sustained binocular stability, reduced recurrence of exotropia, and improved vision-related quality of life. The inclusion of standardized control metrics and patient-reported outcome measures, such as the Intermittent Exotropia Questionnaire (IXTQ), may further clarify the functional and psychosocial impact of postoperative orthoptic rehabilitation in children with intermittent exotropia [12].

## 5. Conclusions

In children undergoing surgical correction for IXT, postoperative OT was associated with substantial short-term improvements in sensory–motor function, particularly in positive fusional vergence and convergence capacity. These findings indicate a meaningful enhancement of binocular control following surgery.

In contrast, changes in static ocular alignment after OT were small and of limited clinical relevance. When final postoperative alignment was compared cross-sectionally between children who underwent OT and those managed with routine follow-up alone, no significant difference in residual deviation was observed. Although this comparison should be interpreted cautiously due to the limited size and heterogeneity of the non-OT group, it suggests that children referred for postoperative OT—who typically present with greater sensory–motor impairment—achieve final alignment comparable to their non-treated peers.

Overall, these results support the role of postoperative OT as a functional adjunct to strabismus surgery rather than a modality aimed at modifying surgical motor outcomes. OT appears to consolidate binocular sensory–motor integration and strengthen functional control, without compromising postoperative ocular alignment.

Future prospective randomized studies with longer follow-up periods are required to determine the durability of these functional improvements and their potential impact on long-term alignment stability, recurrence rates, and vision-related quality of life in children with IXT.

## Figures and Tables

**Table 1:** Baseline clinical characteristics of patients with and without postoperative OT. † *p*-values are reported for descriptive comparison only. Independent-samples *t*-tests were performed with Welch correction when variance homogeneity was not assumed.

Variable	OT Group (n = 90)	Non-OT Group (n = 160)	*p*-Value †
Age atsurgery (years), mean ± SD	8.21 ± 4.12	9.07 ± 15.13	0.498
Surgical dose (mm), mean ± SD	14.69 ± 2.37	13.19 ± 2.53	<0.001
Residual deviationaftersurgery/before OT (PD), mean ± SD	−7.03 ± 6.943	−1.58 ± 5.84	<0.001
Final postoperative deviation(PD), mean ± SD	−5.22 ± 6.60	−8.00 ± 19.29	0.827

Baseline characteristics of matched participants included in the propensity-score analysis. SD = standardized mean difference. Baseline sensory–motor variables are not included, as these assessments were only performed in patients undergoing OT.

**Table 2:** Pre- and post-orthoptic therapy changes in motor and sensory–motor outcomes. Values are presented as mean ± standard deviation. Paired-samples analyses were performed to assess within-subject changes following OT. Effect sizes were calculated using Cohen’s d. PD: prism diopters.

Outcome Measure	Pre-OT (Mean ± SD)	Post-OT (Mean ± SD)	Mean Change (±SD)	*p*-Value	Cohen’s d
Angleofdeviation (PD)	−7.59 ± 7.00	−5.32 ± 7.15	+2.27 ± 6.46	0.0012	0.35
Nearpointofconvergence (cm)	9.45 ± 4.13	8.15 ± 1.84	−1.30 ± 3.58	0.0009	−0.36
Positive fusional amplitude (°)/(PD)	4.17 ± 4.76/ 7.29 ± 8.33	12.68 ± 5.29/ 22.19 ± 9.25	+8.51 ± 5.64 14.89 ± 9.87	<0.001	1.51
Distance convergence amplitude (PD)	7.30 ± 8.33	22.19 ± 9.26	+14.89 ± 9.87	<0.001	1.51
Near convergence amplitude (PD)	10.95 ± 12.50	33.29 ± 13.89	+22.34 ± 14.80	<0.001	1.51

## Data Availability

The datasets generated and analyzed during the current study are not publicly available due to privacy reasons but are available from the corresponding author on reasonable request.
